# BRG1 Is a Prognostic Marker and Potential Therapeutic Target in Human Breast Cancer

**DOI:** 10.1371/journal.pone.0059772

**Published:** 2013-03-22

**Authors:** Jin Bai, Pengjin Mei, Cuipeng Zhang, Feifei Chen, Chen Li, Zhenqiang Pan, Hui Liu, Junnian Zheng

**Affiliations:** 1 Jiangsu Key Laboratory of Biological Cancer Therapy, Xuzhou Medical College, Xuzhou, Jiangsu, China; 2 The Affiliated Hospital of Xuzhou Medical College, Xuzhou, Jiangsu, China; 3 School of Pathology, Xuzhou Medical College, Xuzhou, Jiangsu, China; 4 Department of Oncological Sciences, Mount Sinai School of Medicine, New York, New York, United States of America; University of Bonn, Institut of Experimental Hematology and Transfusion Medicine, Germany

## Abstract

BRG1, a core component of the SWI/SNF chromatin-remodeling complex, has been implicated in cancer development; however, the biological significance of BRG1 in breast cancer remains unknown. We explored the role of BRG1 in human breast cancer pathogenesis. Using tissue microarray and immunohistochemistry, we evaluated BRG1 staining in 437 breast cancer specimens and investigated its role in breast cancer cell proliferation, migration and invasion. Our Kaplan-Meier survival curves showed that high BRG1 expression is inversely correlated with both overall (*P* = 0.000) and disease-specific (*P* = 0.000) 5-year patient survival. Furthermore, we found that knockdown of BRG1 by RNA interference markedly inhibits cell proliferation and causes cessation of cell cycle. This reduced cell proliferation is due to G1 phase arrest as cyclin D1 and cyclin E are diminished whereas p27 is upregulated. Moreover, BRG1 depletion induces the expression of TIMP-2 but reduces MMP-2, thereby inhibiting the ability of cells to migrate and to invade. These results highlight the importance of BRG1 in breast cancer pathogenesis and BRG1 may serve as a prognostic marker as well as a potentially selective therapeutic target.

## Introduction

Breast cancer is a leading cause of death among women worldwide, with approximately 400, 000 deaths per year [1]. The high mortality is attributed, at least in part, to complications of tumor dissemination and distant metastasis. Metastasis is a multistep process requiring tumor cell growth, migration, intravasation, survival in circulation, extravasation and colonization to a secondary site [2]. Therefore, interrupting the metastatic process is of key importance to decrease breast cancer mortality. Alterations in chromatin play an important role in breast cancer progression and metastasis, but the exact molecular mechanisms remains elusive [3].

Cellular transformation is the characteristic of cancer development and progression. The primary cause for cellular transformation is aberrant expression of genes that are involved in cell proliferation, migration, invasion and survival. Gene transcriptional regulation is controlled by the chromatin remodeling complexes. The balance of chromatin remodeling activities may be crucial to ensure accurate responses to developmental or environmental cues and to prevent the transition of normal cells into cancer cells [4]. SWI/SNF chromatin-remodeling complex contributes to epigenetic regulation by utilizing the energy of ATP hydrolysis to remodel chromatin and regulate transcription of target genes, thereby controlling many cellular processes that include DNA repair [5], [6]. This complex is a 1.5 to 2.0-MDa multi-subunit complex, which was first identified in yeast and is highly conserved among eukaryotes. SWI/SNF contains one of two related ATPases, BRG1 or BRM, and 9–12 associated factors (BAFs) [7]. BRG1, BRM and other components of the SWI/SNF complex have been implicated in cancer development. Mice heterozygotes for BRG1 are susceptible to neoplasia and display large subcutaneous tumors [8]. BRG1 or BRM expression is decreased in a wide array of tumors and human cancer cell lines [9], [10]. Loss of both BRG1 and BRM expression correlates with poor prognosis of non-small cell lung cancer [11]. These findings suggest that SWI/SNF functions as a tumor suppressor.

In human cancers, reduced BRG1 expression was found in selected cancer cell lines, and to play a role in the regulation of cellular proliferation [9], [12]. BRG1 binds to retinoblastoma (RB) was shown to repress the activity of E2F1, inhibit the transcription of cyclin A and cyclin E, and mediate G1 arrest [13]. BRG1 can also act upstream of RB by activating the expression of several cyclin-dependent kinase inhibitors (p15, p16 or p21), which leads to the inhibition of CDK2 and CDK4 and accumulation of the hypophosphorylated form of RB that mediates G1 arrest [14]. However, Lin et al. and our group found that knockdown of BRG1 resulted in significantly reduced cell proliferative ability, and this reduced cell proliferation is due to G1 arrest as cyclin D1 is downregulated [15], [16]. Moreover, Naidu et al. showed that BRG1 cooperates with a histone acetyltransferase to constrain p53 activity and permit cancer cell proliferation [17]. Increased BRG1 expression was found in gastric cancer [18], prostate cancer [19], melanoma [16] and glioma [15]. Further studies suggested that higher levels of BRG1 had also been associated with tumor invasiveness.

The role of BRG1 in breast cancer is not well understood. In this article, we sought to investigate the role of BRG1 expression in human breast cancer progression and patient survival and to determine whether this molecule can be used as a prognostic marker and therapeutic target for malignant breast cancer. We used tissue microarray (TMA) technology and immunohistochemistry to evaluate the BRG1 expression level in breast cancer biopsies at different stages. In addition, we further investigated the role of BRG1 in breast cancer cell proliferation, migration and invasion.

## Materials and Methods

### Ethics Statement

This study was performed under a protocol approved by the Institutional Review Boards of The First Affiliated Hospital of Nanjing Medical University and all examinations were performed after obtaining written informed consents.

### Patient Specimens

The study material consists of a series of 437 cases of breast carcinoma aged between 24 and 88 years at the time of diagnoses, from the Departments of Pathology of The First Affiliated Hospital of Nanjing Medical University, between 1996 and 2005. The patients’ clinicopathologic information including age at diagnosis, tumor size, lymph node metastasis, histology grade, histology type, ER status, PR status and HER2 status was obtained from the archive of the pathology department and confirmed by the medical record of the hospital. The histologic grade was assessed using Bloom-Richardson classification. [20] Five-year clinical follow-up results were available for 204 patients.

### Immunohistochemistry

TMA slides were dewaxed at 55°C for 20 min followed by three 5-min washes with xylene. The tissues were then rehydrated by washing the slides for 5-min each with 100%, 95%, 80% ethanol and finally with distilled water. The slides were then heated to 95°C for 30 min in 10 mmol/L sodium citrate (pH 6.0) for antigen retrieval and then treated with 3% hydrogen peroxide for 1 h to block the endogenous peroxidase activity. After blocking the slides with the universal blocking serum, the sections were incubated overnight with monoclonal rabbit anti-BRG1 antibody (1∶100 dilution; Santa Cruz Biotechnology, Santa Cruz, CA, USA) at 4°C. The sections were then incubated for 30 min with a biotin-labeled secondary antibody and then with streptavidin-peroxidase (Zhongshan Biotech, Beijing, China). The samples were developed by treatment with 3, 3′-diamino-benzidine substrate (Zhongshan Biotech, Beijing, China) and with hematoxylin to counter stain the nuclei. Negative controls were done by omitting the BRG1 antibody during the primary antibody incubation.

### Evaluation of Immunostaining

The evaluation of BRG1 staining was done blindly by two pathologists simultaneously, using a multiple viewing microscope. BRG1 staining intensity was scored 0 to 3 (0 = negative; 1 = weak; 2 = moderate; 3 = strong). The percentage of BRG1 positive stained cells was also scored into 4 categories: 1 (0–25%), 2 (26%–50%), 3 (51%–75%), and 4 (76%–100%). The level of BRG1 staining was evaluated by immunoreactive score (IRS) [21], which is calculated by multiplying the scores of staining intensity and the percentage of positive cells. Based on the IRS, BRG1 staining pattern was defined as negative (IRS: 0), weak (IRS: 1–4), moderate (IRS: 6–8), and strong (IRS: 9–12).

### Cell Culture and Transfections

Two human breast carcinoma cell lines MDA-MB-231 and BT-549 were purchased from the Shanghai Institute of Biochemistry and Cell Biology, Chinese Academy of Sciences (Shanghai, China). Cells were cultured in RPMI 1640 medium supplemented with 10% fetal bovine serum (Invitrogen, Shanghai, China). All cells were maintained in 5% CO_2_ atmosphere at 37°C. Cells were grown to 50% confluence before small interfering RNA (siRNA) transfection. Nonspecific control siRNA or BRG1 siRNA (Qiagen, Mississauga, ON, Canada) was transfected by siLentFect Lipid Reagent (Bio-Rad, Hercules, CA, USA) according to the manufacturer’s instructions. The sequence of BRG1 siRNA is: CCG CGC TAC AAC CAG ATG AAA. Twelve hours after transfection, the medium containing transfection reagents was removed. The cells were rinsed twice with phosphate-buffered saline (PBS) and incubated in fresh medium. Cells were lysed for Western blot assay, and subjected to CCK-8 cell proliferation assay, cell migration assay, matrigel invasion assay and cell cycle analysis after transfection.

### Western Blot Analysis

Western blots were performed as previously described [22]. For each treatment group, three parallel samples were applied, and equal amounts of proteins from the parallel samples were mixed and used for blots. The following antibodies were used for Western blot: mouse anti-BRG1, mouse anti-Cul1 (Santa Cruz Biotechnology, Santa Cruz, CA), rabbit anti-cyclin D1, mouse anti-cyclin E, mouse anti-p27, rabbit anti-TIMP-2, rabbit anti-MMP-2 (all from Cell Signaling Technology, Beverly, MA, USA), and mouse anti-β-actin (Boster Biotechnology, Wuhan, China). Each blot was repeated three times.

### Cell Proliferation Assay

Cellular proliferation was analyzed using a WST-8 Cell Counting Kit-8 (Beyotime, Nantong, China). 3×10^3^ cells suspended in 100 µl RPMI 1640 medium containing 10% fetal bovine serum were seeded in 96-well plates and incubated for 24 h, 48 h, 72 h and 96 h. 10 µl CCK-8 solution was added to each well and the cultures were incubated at 37°C for 1 h. Absorbance at 450 nm was measured on an ELX-800 spectrometer reader (Bio-Tek Instruments, Winooski, USA).

### Cell Cycle Analysis

Cells were transfected with nonspecific control siRNA or BRG1 siRNA for 36 h and then treated with 1 µg/ml aphidicolin. Twelve hours after treatments, the medium containing aphidicolin was removed. The cell was rinsed with PBS and then incubated in fresh medium containing 50 ng/ml nocodazole for 0 h, 3 h, 6 h and 9 h. Then cells were fixed with 70% cold ethanol at 4°C overnight, and stained with 40 µg/ml propidium iodide in hypotonic fluorochrome buffer (0.1% Triton X-100, 0.1% sodium citrate, and 25 µg/ml RNase A) for 30 min. Samples were then analyzed using a FACSCanto flow cytometer (BD Biosciences, San Jose, CA, USA). Cell distribution in the different phases of the cell cycle was analyzed with ModFit LT 3.0 software.

### Cell Migration Assay

Cell migration was determined by using a modified two-chamber migration assay with a pore size of 8 µm. For migration assay, 1×10^5^ cells suspended in 200 µl of serum-free medium were seeded on the upper compartment of 24-well Transwell culture chamber, and 600 µl of complete medium was added to the lower compartment. After 12 h incubation at 37°C, cells were fixed with methanol. Non-traversed cells were removed from the upper surface of the filter carefully with a cotton swab. Traversed cells on the lower side of the filter were stained with crystal violet and counted.

### Cell Invasion Assay

The invasion assay was performed using a modified two-chamber plates with a pore size of 8 µm. For invasion assay, 30 µl of 50 mg/ml Matrigel (BD Biosciences, Mississauga, Canada) in serum-free medium was added to the upper compartment of 24-well Transwell culture chamber. 1×10^5^ cells suspended in 200 µl of serum-free medium were seeded on the upper compartment, and 600 µl of complete medium was added to the lower compartment. After 24 h incubation at 37°C, cells were fixed with methanol. Non-invaded cells were removed from the upper surface of the filter carefully with a cotton swab. Invaded cells on the lower side of the filter were stained with crystal violet and counted.

### Gelatin Zymography

2×10^6^ cells were seeded in 100-mm plate for 24 h, cells were transfected with nonspecific control siRNA or BRG1 siRNA. Thirty-six hours after transfection, serum-free medium was applied to the cells overnight and the proteins in the conditioned medium were concentrated with Ultracel-30 k centrifugal filters (Millipore, Billerica, MA) at 5, 000×*g* for 20 min at 4°C. Proteins (50 µg) were loaded on a 10% polyacrylamide gel containing 0.1% gelatin (Sigma, St Louis, MO, USA). After electrophoresis, gel was incubated in Triton X-100 exchange buffer (20 mM Tris-HCl [pH 8.0], 150 mM NaCl, 5 mM CaCl_2_ and 2.5% Triton X-100) for 30 min followed by 10 min wash with incubation buffer (same buffer without Triton X-100) thrice. The gel was then incubated in incubation buffer overnight at 37°C, stained with 0.5% Coomassie blue R250 (Sigma) for 4 h and destained with 30% methanol and 10% glacial acetic acid for 2 h. Gelatinolytic activity was shown as clear areas in the gel.

### Statistical Analysis

Statistical analysis was performed with SPSS 20.0 software (SPSS, Chicago, IL). Data are expressed as the means ± SD. The association between BRG1 staining and the clinicopathologic parameters of the breast cancer patients, including age, tumor size, lymph node metastasis, histology grade, histology type, ER status, PR status and HER2 status, was evaluated by χ^2^ test. The Kaplan-Meier method and log-rank test were used to evaluate the correlation between BRG1 expression and patient survival. Univariate or multivariate Cox proportional hazards regression models were performed to estimate the crude hazard ratios (HRs) or adjusted HRs and their 95% confidential intervals (CIs). For CCK-8 cell proliferation assays, Student *t* test was used. Differences were considered significant when *P*<0.05.

## Results

### Correlation of BRG1 Staining with Clinicopathologic Parameters and Patient Survival

**Figure 1:**
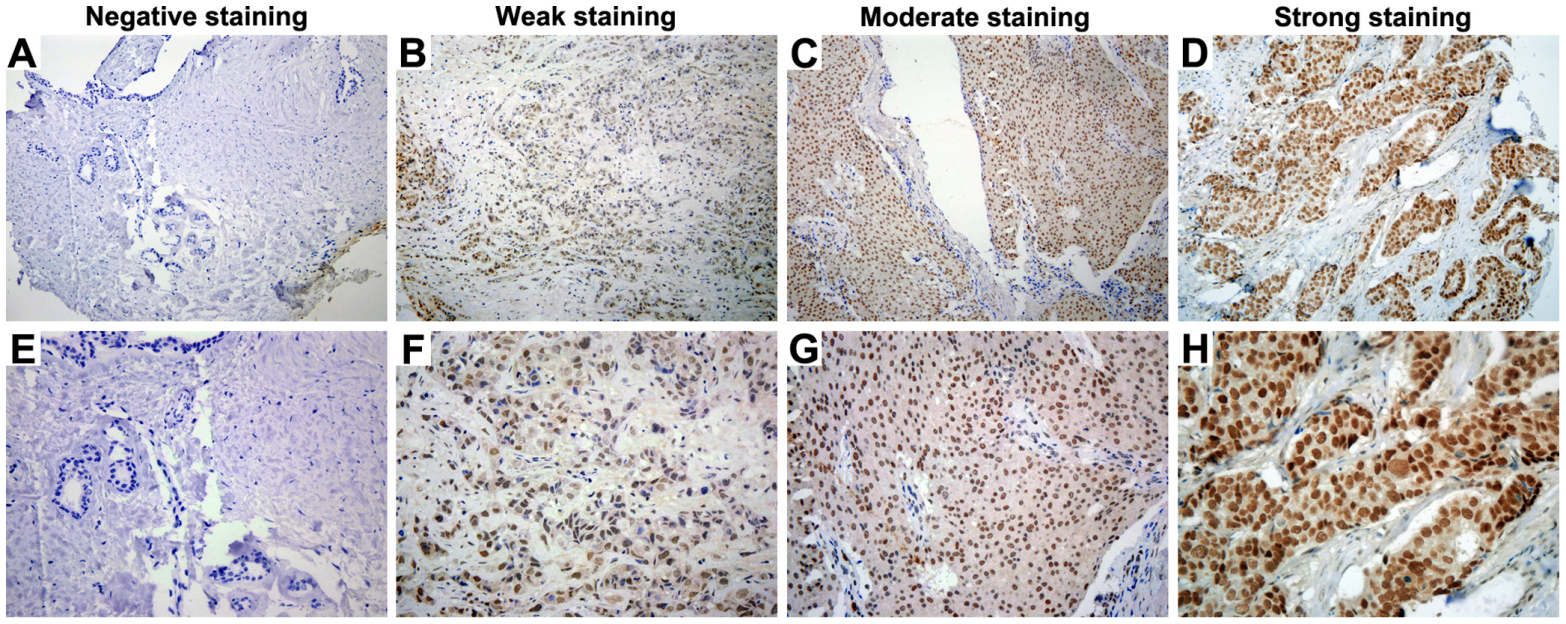
Representative images of BRG1 immunohistochemical staining in human breast cancer. A, E Negative staining. B, F Weak positive staining. C, G Moderate positive staining. D, H Strong positive staining. Magnification×200 for A, B, C, and D; ×400 for E, F, G, and H.

**Figure 2:**
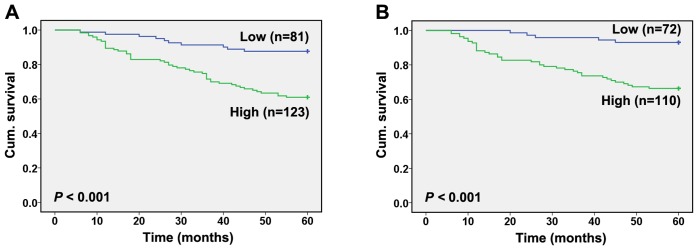
Kaplan-Meier survival analyses of breast cancer patients. A High BRG1 expression correlates with a poorer overall survival (*P*<0.001, log-rank test). B High BRG1 expression correlates with a poorer disease-specific 5-year survival (*P*<0.001, log-rank test). Cum, cumulative.

To monitor BRG1 expression in breast cancer, immunohistochemistry staining was performed in TMA slide containing 437 breast cancer biopsies. The distribution of clinicopathologic features is detailed in Table 1. The immunohistologic staining was classified as negative, weak positive, moderate positive and strong positive (Fig. 1). Of the 437 breast cancer analyzed, low expression levels (negative and weak) and high expression levels (moderate and strong) were 52.4% (229/437) and 47.6% (208/437), respectively. The Kaplan-Meier analysis and log-rank test revealed that increased BRG1 expression was associated with poor overall or disease-specific 5-year patient survival (*P* = 0.000 and *P = *0.000, respectively, log-rank test; Fig. 2A and B). However, no significant correlations were found between BRG1 expression and clinicopathologic variables that include patient age, tumor size, lymph node metastasis, histology grade, histology type, ER status, PR status and HER2 status (Table 1).

**Table 1:** BRG1 staining and clinicopathological characteristics of 437 breast cancer patients.

Variables	BRG1 staining			
	Low (%)	High (%)	Total	*P* *
**Age**				
≤50 years	110 (52.9)	98 (47.1)	208	0.849
>50 years	119 (52.0)	110 (48.0)	229	
**Tumor size**				
T1 (<2 cm)	50 (59.5)	34 (40.5)	84	0.376
T2 (2–5 cm)	158 (51.0)	152 (49.0)	310	
T3 (>5 cm)	16 (51.6)	15 (48.4)	31	
**Lymph node metastasis**				
Negative	108 (53.7)	93 (46.3)	201	0.921
Positive	107 (53.0)	95 (47.0)	202	
**Histology Grade**				
I	20 (50.0)	20 (50.0)	40	0.753
II	127 (54.3)	107 (45.7)	234	
III	38 (50.0)	38 (50.0)	100	
**Histology Type**				
Ductal	204 (53.0)	181 (47.0)	385	0.670
Lobular	15 (53.6)	13 (46.4)	28	
other	10 (43.5)	13 (56.5)	23	
**ER status**				
Negative	38 (45.8)	45 (54.2)	83	0.773
Positive	50 (43.1)	66 (56.9)	116	
**PR status**				
Negative	50 (51.0)	48 (49.0)	98	0.064
Positive	38 (37.6)	63 (62.4)	101	
**HER2 status**				
Negative	13 (39.4)	20 (60.6)	33	0.561
Positive	63 (46.0)	74 (54.0)	137	

* *P* values are from χ^2^ test.

We also used univariate Cox proportional hazards regression model to estimate the crude hazard ratios (HRs) of BRG1 expression or each clinicopathological variable on patient survival. The log-rank test and univariate Cox regression analyses revealed BRG1 expression were significantly associated with overall (*P* = 0.005) or disease-specific (*P* = 0.003) survival in breast cancer patients (Table 2). To further validate the prognostic value of BRG1, multivariate analysis was performed and significant factors are summarized in Table 3. The Cox regression model indicated that expression of BRG1 is an independent prognostic marker for both overall (*P* = 0.018) and disease-specific survival (*P* = 0.026).

**Table 2:** Univariate Cox proportional regression analysis on 5-year overall and disease-specific survival of 437 breast cancer patients.

Variable*	Overall survival			Disease-specific survival					
	Hazard ratio	95% CI†	*P* *	Hazard ratio		95% CI†		*P* *	
BRG1									
Low expression	1.000		0.005	1.000				0.003	
High expression	2.720	1.694–4.363		2.414		1.442–4.027			
Age									
≤50 years	1.000		0.595	1.000				0.963	
>50 years	1.138	0.731–1.746		1.001		0.620–1.648			
Tumor size									
≤5 cm	1.000		0.009	1.000				0.008	
>5 cm	2.361	1.509–4.178		2.495		1.671–4.646			
Lymph node metastasis									
Negative	1.000		0.000	1.000				0.000	
positive	4.491	2.637–7.70		4.105		2.492–6.753			
Histology Grade									
I	1.000		0.000	1.000				0.000	
II/III	3.290	2.081–5.193		3.762	2.382–5.939				
Histology type									
Ductal	1.000		0.322	1.000			0.158		
Lobular and other	1.224	0.824–1.805		1.347	0.896–2.002				

* *P* values are from Log-rank test.

† CI: confidence interval.

**Table 3:** Multivariate Cox regression analysis on 5-year overall and disease-specific survival of 437 breast cancer patients.

Variable*	Overall survival			Disease-specific survival		
	Hazard ratio	95% CI†	*P*	Hazard ratio	95% CI	*P*
BRG1	1.847	1.116 to 3.065	0.018	1.657	0.930 to 2.761	0.026
Age	1.219	0.765 to 1.906	0.420	1.134	0.687 to 1.896	0.629
Tumor size	1.456	0.889 to 2.396	0.145	1.480	0.851 to 2.583	0.102
Lymph node metastasis	2.406	1.462 to 3.958	0.000	2.331	1.368 to 3.764	0.000
Histology Grade	2.057	1.230 to 3.425	0.006	2.179	1.246 to 3.803	0.003
Histology type	0.905	0.621 to 1.328	0.589	0.891	0.545 to 1.207	0.289

* Coding of variables: BRG1 was coded as 1 (low expression), and 2 (high expression). Age was coded as 1 (≤50 years), and 2 (>50 years). Tumor size was coded as 1 (≤5 cm), and 2 (>5 cm). Lymph node metastasis was coded as 1 (negative), and 2 (positive). Histology grade was coded as 1 (I), and 2 (II and III). Histology type was coded as 1 (dutcal), and 2 (lobular and other).

† CI: confidence interval.

### BRG1 Regulates Breast Cancer Cells Proliferation and Cell Cycle

**Figure 3:**
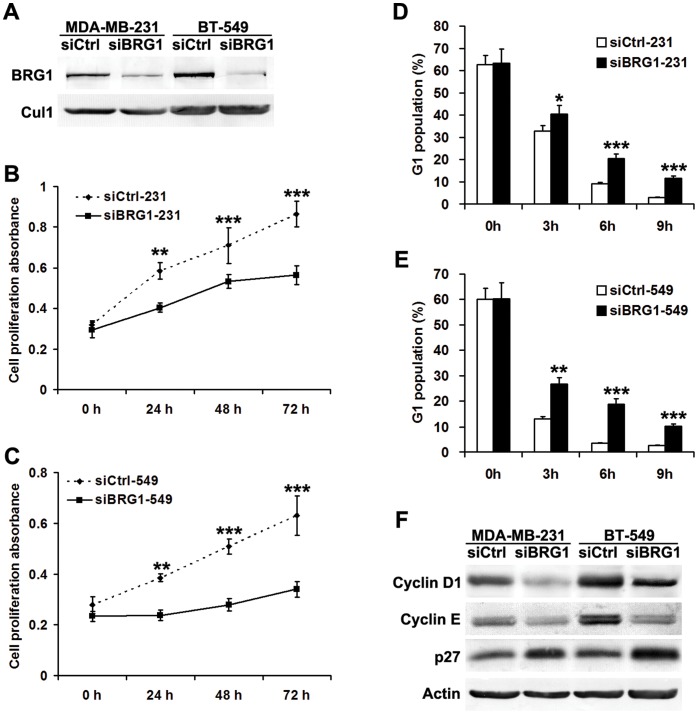
BRG1 knockdown reduces breast cancer cells proliferative ability. A Forty-eight hours after transfection, the expression of BRG1 in MDA-MB-231 and BT-549 cells was evaluated by Western blot. Cul1 was used as a nucleoprotein loading control. B, C CCK-8 cell proliferation assay was performed after BRG1 knockdown in MDA-MB-231 and BT-549. D, E The percentage of G1 population cells was measured by flow cytometry after BRG1 knockdown in MDA-MB-231 and BT-549 cells. F Western blot analysis of the relative protein levels of cyclin D1, cyclin E and p27 in BRG1 knockdown and control group of MDA-MB-231 and BT-549 cells. β-Actin was used as a whole cell protein loading control. All experiments were carried out in triplicate. Data are shown as mean ± SE. ** *P*<0.01; *** *P*<0.001.

Since increased BRG1 expression is associated with poor prognosis, BRG1 may play important roles in tumor development. We first transiently transfected MDA-MB-231 and BT-549 human breast cancer cells with BRG1 siRNA or control siRNA. Forty-eight hours after transfection, cells were harvested for Western blot analysis (Fig. 3A) or subjected to cell proliferation assays. Western blot results confirmed significant reduction of BRG1 in either MDA-MB-231 or BT-549 cells transfected with BRG1 siRNA. The results of CCK-8 cell proliferation assays revealed slowed growth rates in either MDA-MB-231 or BT-549 breast cancer cells depleted of BRG1 (Fig. 3B and C).

To determine if the reduced cell proliferation of BRG1 knockdown cells is due to cell cycle arrest, we performed flow cytometry analysis. The results showed that knocking down BRG1 in either MDA-MB-231 or BT-549 cells resulted in an increase of cell population at G1 phase (Fig. 3D and E). Moreover, immunoblot analysis showed increased p27 expression but decreased levels of cyclin D1 or cyclin E (Fig. 3F) in breast cancer cells that lacked BRG1.

### Silencing of BRG1 Inhibits Breast Cancer Cells Migration and Invasion in vitro

**Figure 4:**
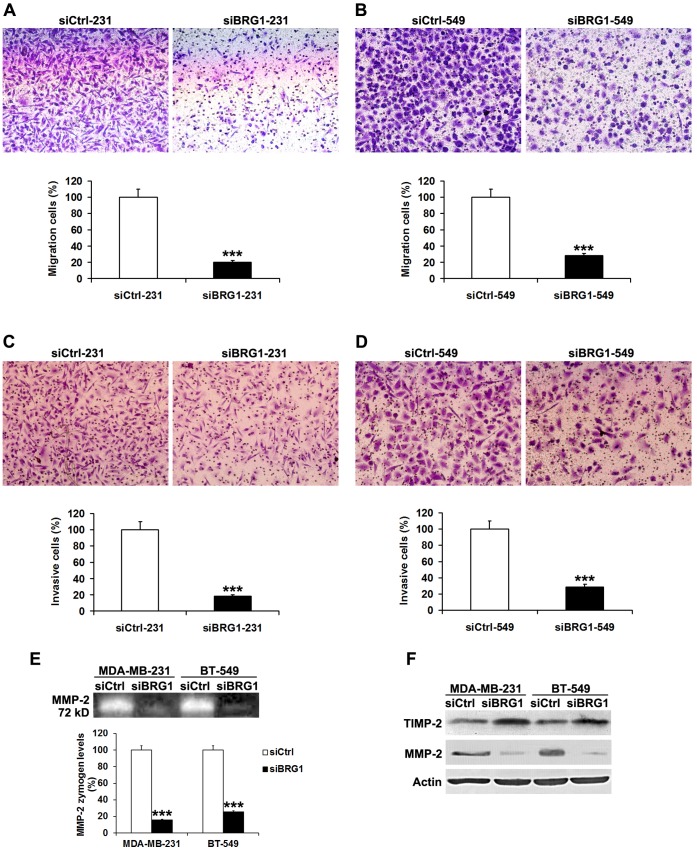
Knockdown of BRG1 inhibits breast cancer cell migration and invasion. A, B Cell migration assay was performed after the knockdown of BRG1 in MDA-MB-231 and BT-549 cells. C, D Matrigel cell invasion assay was performed after the knockdown of BRG1 in MDA-MB-231 and BT-549 cells. E Gelatin zymography analysis of the relative enzyme activities of MMP-2 in BRG1 knockdown and control siRNA group for both MDA-MB-231 and BT-549 cell lines. F Western blot analysis of the relative protein levels of TIMP-2 and MMP-2 in BRG1 knockdown and control siRNA group for both MDA-MB-231 and BT-549 cell lines. All experiments were carried out in triplicate. Data are shown as mean ± SE. *** *P*<0.001.

We next investigated the role of BRG1 in migration and invasion of breast cancer cells. The results of cell migration assay showed that BRG1 knockdown decreased cells migration ability of MDA-MB-231 and BT-549 cells by 79% and 68%, respectively (Fig. 4A and B). Furthermore, silencing of BRG1 inhibited the invasive ability of MDA-MB-231 and BT-549 cells by 81% and 72%, respectively (Fig. 4C and D).

We performed gelatin zymography to measure the MMP-2 and MMP-9 activities and Western blot to examine the TIMP-1, TIMP-2, MMP-2 and MMP-9 expressions in breast cancer cells. The MMP-2 enzyme activity was significantly suppressed after knockdown of BRG1 in MDA-MB-231 and BT-549 cells, exhibiting 16% and 25% of the control level, respectively (Fig. 4E). Western blot results showed that inhibition of MMP-2 is correlated to increased expression of TIMP-2 in MDA-MB-231 and BT-549 cells lacking BRG1 (Fig. 4F).

## Discussion

In eukaryotic cells, DNA is packaged into chromatin and this compact state contributes to transcriptional repression. Chromatin remodeling complexes are responsible for making DNA accessible to transcription factors and therefore, actively participate in gene expression [4]. The mammalian SWI/SNF complex mediates ATP-dependent chromatin remodeling processes that are critical for transcriptional regulation [5], [6], [9], [23]. Increasing evidence has indicated a role for inactivation of members of SWI/SNF complex including BRG1, BRM, SNF5, BAF155 and BAF57 in cancer development and/or cancer progression [24]. The BRG1 and BRM are concomitantly lost in 15–20% of primary non-small cell lung carcinomas, which was closely correlated with poor prognosis [11]. Loss of the expression of BRG1 is frequently observed in intraductal papillary mucinous neoplasms of the pancreas [25]. Increased expression of BRG1 was found in gastric cancer [18], prostate cancer [19], colorectal carcinoma [26], glioma [15] and melanoma [16]. However, the expression of BRG1 in breast cancer is poorly defined. In this study, we used TMA technology and immunohistochemistry to investigate BRG1 expression in 437 cases of human breast cancer. Our Kaplan-Meier analyses demonstrated that increased BRG1 expression is significant correlated with a poorer 5-year overall and disease-specific patient survival in breast cancer, suggesting that elevated BRG1 expression may serve as a molecular prognostic marker for this disease.

Many studies suggested BRG1 as a tumor suppressor. BRG1 is found inactivated in many human cancers and cell lines. It interacts with tumor suppressors such as RB and its family members, LKB1 and HIC1, and this interaction may have a role in the repression of E2F-responsive genes and growth suppression [14], [27], [28]. We found marked reduction of cell proliferation and cessation of cell cycle after BRG1 knockdown, presumably as a result of inhibition of cyclin D1 and cyclin E, and increased expression of p27, thereby resulting in cell cycle arrest at the G1 phase. Gene expression data revealed that the arrest may in part be accounted for by downregulation of E2F target genes such as cyclin E and upregulation of the cyclin-dependent kinase inhibitors p21, p15 and p16 [13], [29]. In addition, BRG1 protein directly interacts with BRCA1 tumor suppressor and subsequently stimulates transcriptional activity of the p53 protein [30]. Our results are in agreement with the findings in human melanoma and glioma cell lines [15], [16]. We previously showed that knockdown of BRG1 in glioma cell lines resulted in significantly reduced cell proliferative ability, and this reduced cell proliferation is due to G1 phase arrest as cyclin D1 is downregulated [15]. Furthermore, Keenen et al. found that BRG1 interact with an oncoprotein, the microphthalmiassociated transcription factor (MITF), to promote melanoma proliferation [31]. BRG1 permitted cancer cell proliferation in cooperation with the histone acetyl transferase, CREB-binding protein, to suppress p53 activity [17]. Therefore, it is difficult to conclude whether BRG1 is indeed a tumor suppressor or oncogene. These findings indicate the possibility that the biological significance of BRG1 during the pathogenesis of human cancer differ according to cell and/or tissue type, but the exact molecular mechanism warrants further investigation.

We provided experimental evidence that the expression level of BRG1 is related to breast cancer cell migration and invasion. Tissue invasion is an essential step in metastasis that requires breakdown of the extracellular matrix (ECM) around the cancer cells. Matrix metalloproteinases (MMPs) play a critical role in tumor invasion by cleaving the ECM components [32]. MMP activity is controlled by specific, endogenous tissue inhibitors of metalloproteinases (TIMPs). TIMP-2 is a main negative regulator of MMP-2 enzyme activity and involved in several tumor metastasis processes, including breast cancer [33]. In this study, our data demonstrated that knockdown of BRG1 in breast cancer cells resulted in significantly inhibited cell migration and invasion abilities. Gelatin zymography and western blot showed that BRG1 siRNA inhibited MMP-2 enzyme activity, and this was consistent with the upregulation of TIMP-2. This result is in agreement with the report by Sun et al. demonstrating that overexpression of BRG1 enhances prostate cancer cell invasion [19]. In fact, Saladi et al. found that activation of MMP-2 expression greatly contributed to the BRG1 induced increase in melanoma invasiveness, and BRG1 is recruited to the MMP-2 promoter and directly activates expression of this metastasis associated gene [34].

In summary, we demonstrated that BRG1 plays an important role in human breast cancer pathogenesis. Increased BRG1 expression may facilitate tumor progression by enhancing cell growth, migration and invasion. Our results imply that BRG1 may serve as a prognostic marker as well as a potential therapeutic target for breast cancer.
